# Supplementary material for: An Ultrasensitive Spatial Tissue Proteomics Workflow Exceeding 100 Proteomes Per Day
Source: Mol Cell Proteomics. 2025 Dec 17;25(1):101489. doi: 10.1016/j.mcpro.2025.101489 (PMC12828817; doi:10.1016/j.mcpro.2025.101489)
Supplement: Supplementary Information [file mmc1.docx]

**Supplementary Information**

**An ultrasensitive spatial tissue proteomics workflow exceeding 100 proteomes per day**

Klingeberg et al.

**Supplementary Figures**

**Supplementary Fig. 1, related to Fig. 1:
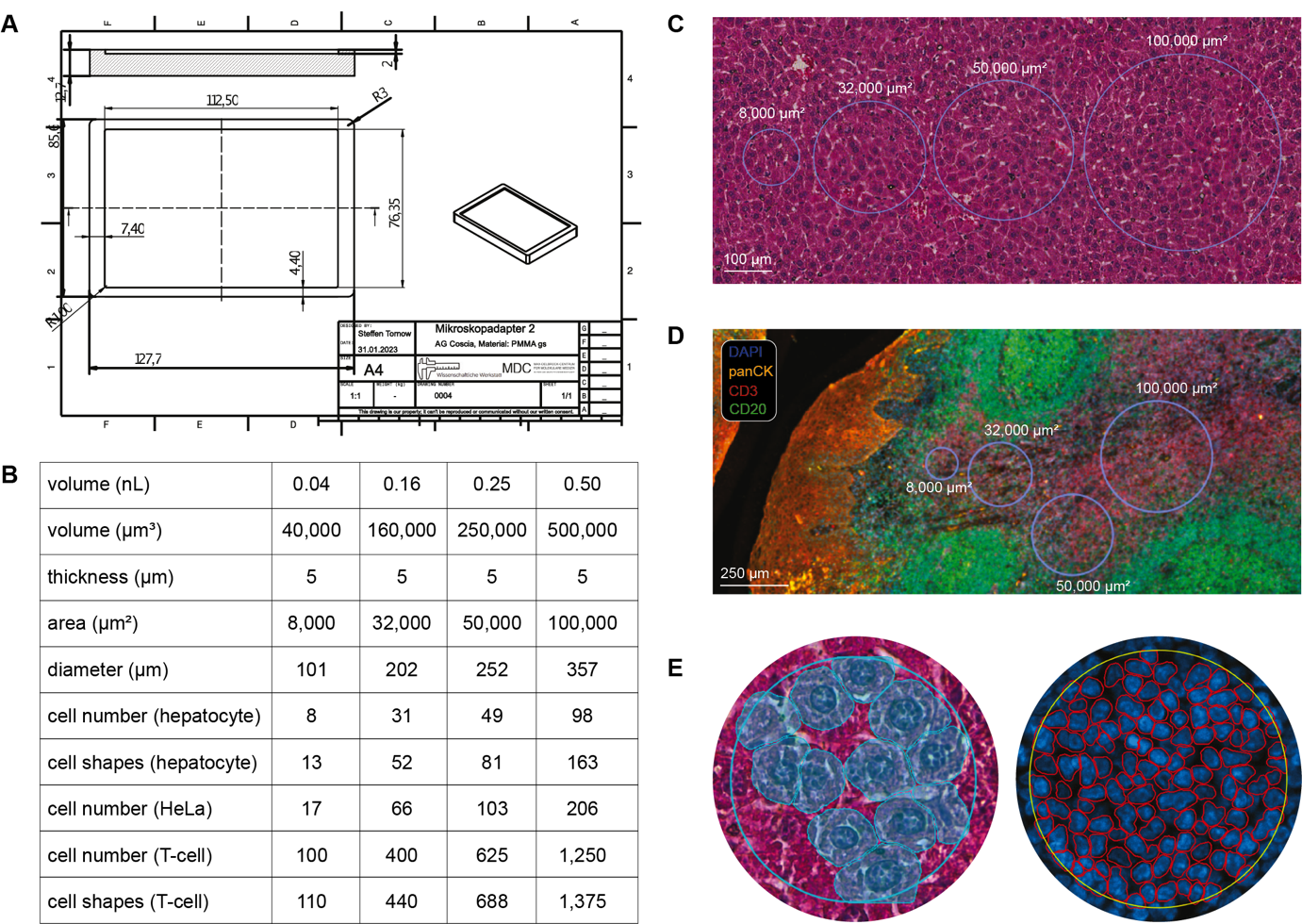
**

(a) Technical drawing of LMD7 adapter compatible with the proteoChip-Evo96. (b) Tissue areas and corresponding volumes for 5 µm-thick tissue sections. Tissue volumes were estimated using the cylindrical approximation (area × thickness). Volumes were converted using the factor: 1 nL = 10⁶ µm³. Approximate cell counts were derived based on average cell volumes of hepatocytes, HeLa cells, and T lymphocytes (https://bionumbers.hms.harvard.edu/search.aspx). Cell shapes were estimated based on segmentation as shown in e. (c) H&E staining of 5 µm murine liver tissue section, illustrating four isolated area sizes: 8,000 µm^2^, 32,000 µm^2^, 50,000 µm^2^, 100,000 µm^2^. (d) Immunofluorescent staining of a 5 µm thick human tonsil tissue section, showing panCK (orange), CD3 (red), CD20 (green) and DAPI (blue). (e) Cell segmentation of murine liver tissue (left) and human tonsil t issue (right) of 8,000 µm^2^.

**Supplementary Fig. 2, related to Fig. 1:**


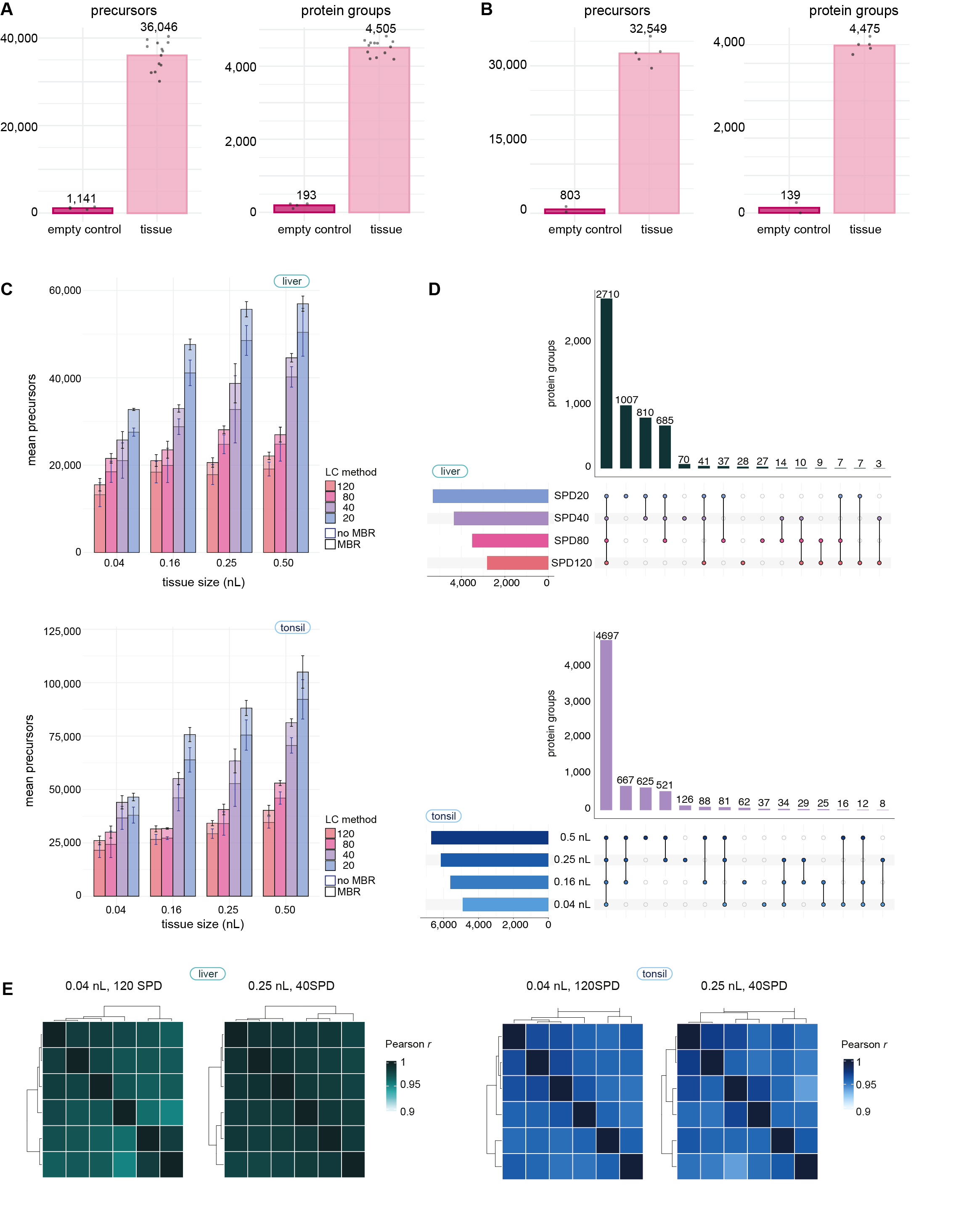


(a, b) Number of identified precursors and protein groups from murine liver tissue of 0.25 nL volume from two independent experiments including negative control samples. (c) Identified precursors from different sized liver (top) and tonsil (bottom) tissue samples measured with different Whisper Zoom methods. Mean values from six to eight replicates are shown, with (blue) and without (black) MBR. (d) UpSet plots showing the overlap of identified proteins across four different LC gradients in 0.25 nL liver tissue (top). Upset plot showing the overlap of identified proteins across four sizes of tonsil tissue measured at 40 SPD (bottom). Each bar represents the number of proteins uniquely or commonly identified between the conditions. Connected dots below the bars indicate which condition(s) contribute to each set. (e) Heatmap of Proteome correlation (Pearson’s r) between replicates of the same size measured with identical LC gradients for liver (left) and tonsil (right).


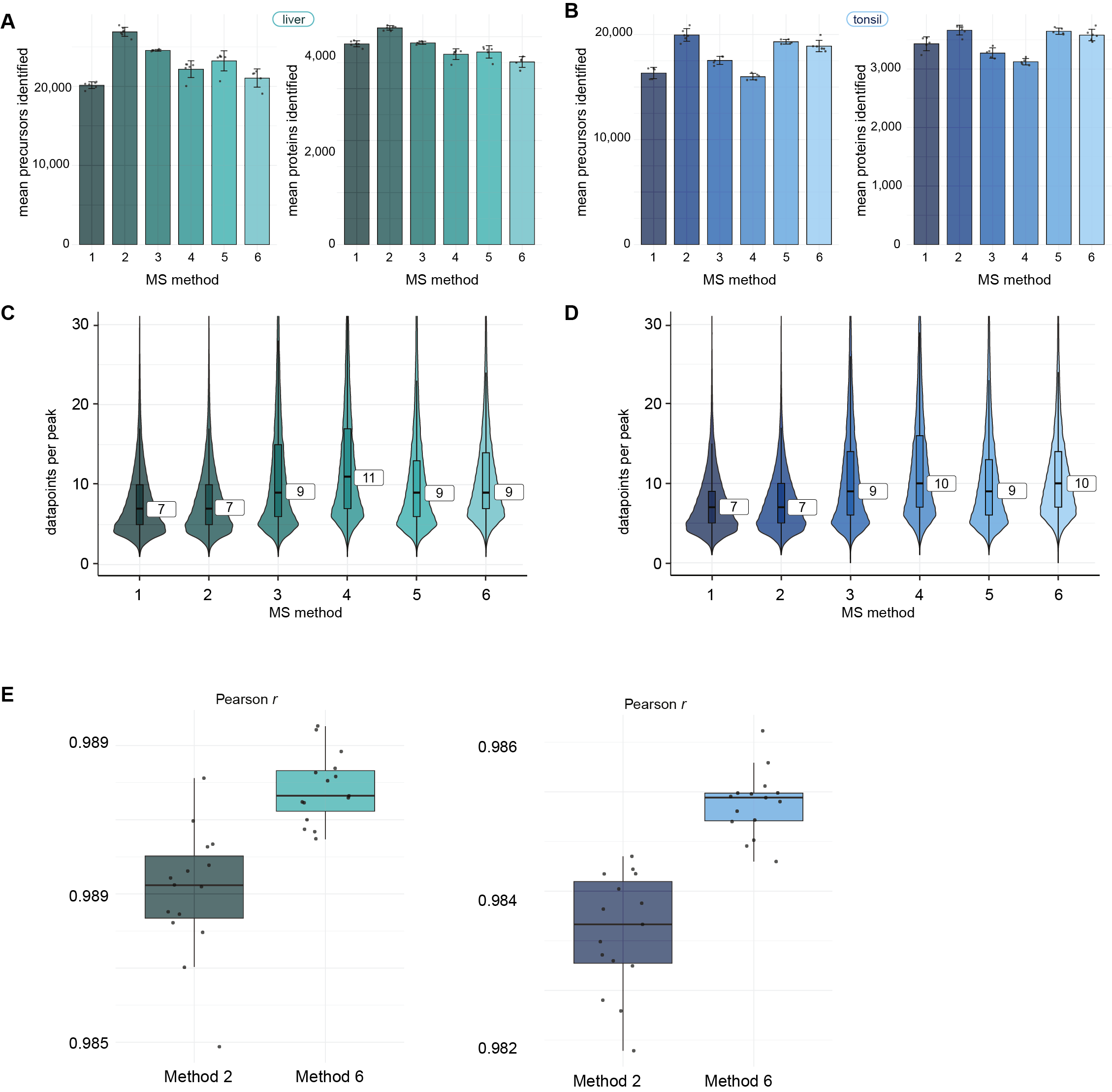
**Supplementary Fig. 3, related to Fig. 2:**

(a) Precursor and protein identification from liver tissue samples measured using different MS methods. Averages are shown from five measurements from one bulk prepapred sample. (b) Precursor and protein identifications from T-cell-rich tonsil tissue samples measured using different MS methods. Averages are shown from five measurements from one bulk prepapred sample. (c, d) Violin plots display the distribution of data points per peak for each MS method in the liver (c) and tonsil (d) tissues. The overlaid boxplots show the median (central line), interquartile range (box), and whiskers extending to 1.5 × the IQR. (e) Boxplots showing proteome correlations (Pearson’s r) for methods #2 and #6.

**Supplementary Tables**

**Supplementary Table 1: Variable isolation window information of pyDIAid optimized method**

This table presents the ion mobility (IM) and mass ranges for the five variable windows of the pyDIAid optimized method.

**Supplementary Table 2: Quantitative coverage of immune system process and T-cell-specific proteins in T-cell enriched proteomes.**

This table presents the quantitative coverage of immune system process proteins (a) and T-cell-specific pathway proteins (b) detected in the whole proteome versus a single 20 SPD injection from a T-cell-enriched tissue sample. The analysis is based on Gene Ontology Biological Process (GOBP) and related annotations. For each functional category, the table reports the total number and percentage of proteins detected in each dataset, along with the corresponding GO term name and detection ratio. These values support the coverage analysis presented in **Fig. 1h** and demonstrate the depth and specificity of immune-related protein profiling.

**Supplementary Table 3: Proteins differentially expressed across tumor, stroma, and immune compartments.**

This table contains the 1,663 proteins identified as significantly different between tissue compartments (tumor, stroma, and immune infiltrate) based on one-way ANOVA analysis. These proteins were used for unsupervised hierarchical clustering to reveal compartment-specific expression profiles, as shown in **Fig. 3e**. For each protein, we provide pathway and functional annotations including Hallmark gene sets, Reactome pathways, WikiPathways, and Gene Ontology terms for Biological Process (GOBP), Molecular Function (GOMF), and Cellular Component (GOCC). Statistical significance is reported as the negative log10-transformed ANOVA *p*-value and the corresponding *q*-value (FDR-adjusted). Protein identifiers (Protein Group, Protein Names, Genes) and detailed descriptions (First Protein Description) are included to facilitate biological interpretation of compartment-specific proteomic signatures.

**Supplementary Table 4: Proteins showing high spatial variability across tumor regions.**

This table presents a comprehensive list of proteins identified from spatially resolved proteomic profiling that exhibit high variability in abundance across different regions within the same tumor. These proteins were selected based on their coefficient of variation, highlighting substantial intratumoral heterogeneity in protein expression. Each entry includes pathway and ontology annotations—Hallmark gene sets, Reactome pathways, WikiPathways, and Gene Ontology terms for Biological Process (GOBP), Molecular Function (GOMF), and Cellular Component (GOCC)—to contextualize the potential functional roles of the variable proteins. Quantitative measures include the mean protein intensity across regions, the global coefficient of variation, and region-specific variability metrics such as Density_Mean_Coefficient of Variation and Excluded Fraction_Mean_Coefficient of Variation. Additional columns report protein identifiers (Protein Group, Protein Names, Genes) and functional descriptions. This dataset underpins the analysis shown in **Fig. 4a**, where proteins with high regional variability are visualized.
